# Three-Dimensional Bi_2_Fe_4_O_9_ Nanocubes Loaded on Reduced Graphene Oxide for Enhanced Electromagnetic Absorbing Properties

**DOI:** 10.3389/fchem.2020.00608

**Published:** 2020-08-07

**Authors:** Min Lu, Yuan-Kai Sun, Shu-Hao Yang, Hui-Ya Wang, Xiao-Hui Guan, Guang-Sheng Wang

**Affiliations:** ^1^Northeast Electric Power University, Jilin, China; ^2^School of Chemistry, Beihang University, Beijing, China

**Keywords:** Bi_2_Fe_4_O_9_(BFO) nanocubes, Bi_2_Fe_4_O_9_/rGO nanohybrids, wave absorption property, a potential EMW material, hydrothermal method, a rather wide frequency band, synergy effect

## Abstract

Bi_2_Fe_4_O_9_(BFO) nanocubes were prepared in proportion using a simple and easy hydrothermal method, and were then assembled on reduced graphene oxide (rGO) multilayered sheets. The excellent microwave absorption properties of Bi_2_Fe_4_O_9_/rGO nanohybrids were achieved by properly adjusting the impedance matching and getting a high attenuation capability contributed from different ratios of the BFO and rGO. A minimum reflection loss value of −61.5 dB at 12.8 GHz was obtained with a Bi_2_Fe_4_O_9_/rGO ratio of 2:1, and the broadest bandwidth below −10 dB was up to 5.0 GHz (from 10.8 to 15.8 GHz) with a thickness of 2.4 mm. Additionally, the elementary mechanism of wave absorption performance is also investigated.

## Introduction

Communication equipment, such as mobile phones and fax machines, have brought great convenience to people. At the same time, because of their electromagnetic radiation pollution, they also bring many hidden dangers. In order to address this issue, electromagnetic (EM) wave absorbing materials have attracted an abundance of attention from various fields (Sun et al., 2014; Li et al., 2018a; Liu et al., 2019; Mo et al., 2019). Magnetic materials and their composites are accepted as one of the most significant EM absorbing materials, owing much to their high EM performance, broad frequency range response, low price, easy preparation, and excellent chemical stability (Zhao et al., 2013; Dhawan et al., 2015; Qiu et al., 2017; Tang et al., 2017). However, traditional magnetic metal absorbing materials (Fe, Co, Ni) with a high complex permeability make it difficult to satisfy the impedance match in materials and free space (Xu et al., 2018a). Unlike common magnetic metals, ferrites with a relatively high Snoek's limit, medium-built saturation magnetization, and coercivity have become a popular new EM wave absorbing material (Rusly et al., 2018; Trana et al., 2019; Zhu et al., 2019). For example, Lee et al. Prepared M-type hexaferrites BaFe_12−x_Co_x_O_19_ (x = 0–2), which were synthesized by a co-precipitation technique, in which relatively high reflection loss (RL) values with the frequency range of 0.1–15 GHz was obtained (Trana et al., 2019). Matori et al. used a mechanical activation high energy ball milling (HEBM) method to study the EM properties of multiferroic BiFeO_3_ composites under temperatures of 700–800°C (Rusly et al., 2018). Although ferrites have shown immense potential as an EM wave absorbing material, single phase ferrite can still not fully meet the characteristics of being light weight, having a thin thickness, strong absorption, wide absorption bandwidth, and environmental stability. Beyond all doubt, ferrites need to be compounded with other materials to further improve their performance in the field of wave absorption.

To date, there are two main strategies to make ferrite excellent microwave absorbing materials (Liu et al., 2020). Firstly, it is possible to introduce dielectric materials into ferrite becoming an EM compound. Lin et al. fabricated flower-like MoS_2_@Bi_2_Fe_4_O_9_ MPs with a quite broad bandwidth of 5.0 GHz and a high reflection loss of −52.3 dB (Lin et al., 2018). Yang et al. have successfully prepared a rGO/porous Bi_2_Fe_4_O_9_ composite via a dissolution-recrystallization/reduction process, which possesses outstanding EM wave absorption properties and a large absorption bandwidth (Dai et al., 2019). Zhang et al. synthesized a rGO/BiFeO_3_ composite with the maximum reflection loss value of −46.7 dB (Gao et al., 2019). Secondly, it is also possible to investigate constructional EM materials with special particle structures and microscopic shapes (Feng et al., 2017; Zhang et al., 2018; Huang et al., 2019). For example, Huang et al. prepared C/CoFe_2_O_4_ nanocomposites with a special porous structures root in eggshell membrane, which was shown to have a brilliant EM absorption capability (Huang et al., 2019). Well-bedded ZnFe_2_O_4_@SiO_2_@rGO core-shell microspheres exhibited an outstanding microwave absorption performance (Feng et al., 2017). Such ferrite matrix materials have shown more excellent EM wave absorption properties than single ferrite due to the particular microstructure and the concept of bonding magnetic loss to dielectric loss.

Inspired by of the vast amount of research on two dimensional (2D) materials, rGO represents the unique characteristics needed to acquire unparalleled chemical, physical, and electronic properties because of the electron confinement in dimensions (Zhu et al., 2010; Wang et al., 2011; Guo H. et al., 2013). Furthermore, the fantastically great specific surface area and positive electrical conductivity makes rGO an extremely ideal material to incorporate with magnetic loss materials to acquire effective regulation of EM parameters (Hummers and Offeman, 1958; Guo H. B. L. et al., 2013; Zhang et al., 2013, 2014; Shen et al., 2018; Prasad et al., 2019). Han et al. created a two-step process which involves a hydrothermal reaction and surface modification to obtain the graphene-wrapped ZnO hollow spheres that exhibit an maximum reflection loss of −45.1 dB (Han et al., 2014). One Co_3_O_4_ nanosheet/rGO composite, which exhibited a reflection loss value of −45.15 dB as well as an effective broad bandwidth of 5.61 GHz, was synthesized (Zhang et al., 2013). Therefore, it can be concluded that the combination of rGO and ferrites with different microstructures is not only able to satisfy the demand of impedance match, which offers an effective way to develop high performance microwave absorbers, but also results in some additional functions which facilitate their practical application in the absorbers.

Separate from the mainstream research on the photocatalysis (Janisse, 2013) and gas sensor properties (Mohapatra et al., 2017) of BFO, this research focuses on the EM wave absorbing ability of BFO. In this work, we prepared an efficient and facile method to synthesize a large amount of three-dimensional BFO nanocubes which easily load on the rGO nanosheets with varying proportions. PVDF was used to function as a disperse matrix due to the synergy effect between dielectric polymer matrixes and parameters that could further improve the EM wave absorption performance of the composites. The exceptional advantages of PVDF, i.e., its flexibility, low weight, and high chemical corrosion resistance, can also benefit the practical applications of the BFO/rGO/PVDF composite absorber (Liu et al., 2015). Moreover, the wave absorption ability of this material has been significantly advanced through the introduction of rGO and the purpose of meeting the optimal impedance match is achieved by changing the ratio of BFO and rGO to adjust the magnetic and dielectric properties of the compound. As expected, an impressive reflection loss of −61.5 dB and a superior frequency band over 5 GHz is achieved by adjusting the proportion of Bi_2_Fe_4_O_9_ and rGO to a certain ratio of 2:1 when the thickness of the absorber is just 2.4 mm. Additionally, we have further investigated the mechanism of EMW absorbing properties as well as the influence of dielectric loss, magnetic loss, and impedance match for the materials. To sum up, the method of synthesizing BFO/rGO nanohybrids is simple and efficient and a great EMW absorbing performance can be achieved with a thin thickness which indicates that the BFO/rGO nanohybrids have great potential in practical applications.

## Experimental

### Fabrication of the Bi2Fe4O9 Nanoparticles and the Bi2Fe4O9/rGO Nanohybrids

Graphene Oxide (GO) was prepared by a modified Hummer's method (Hummers and Offeman, 1958). The synthesis of Bi_2_Fe_4_O_9_ nanocubes was conducted via a simple hydrothermal reaction (Han et al., 2006). Briefly, 125 mmol of Fe(NO_3_)_3_·9H_2_O and Bi(NO_3_)_3_·5H_2_O was added into a 100 mL steel autoclave. Then, a KOH solution of 12 mol/L was slowly poured into the autoclave until 70–80% of its volume stopped. Subsequently, 50 μl of concentrated hydrochloric acid was added into the autoclave. The reaction mixture solution was constantly stirred by the magnetic stirring apparatus for 45 min. After that, the autoclave was placed in the oven at 200°C for 24 h. Finally, the product was washed several times with deionized water and then dried at 60°C in the oven.

The Bi_2_Fe_4_O_9_/rGO nanohybrids were synthesized by a non *in situ* composite method. Firstly, the 40 mg of graphene oxide was put in 60 ml of deionized water with ultrasonic treatment for 2 h to obtain a homogeneous dispersion. Then 525 μl of ammonia and 33 μl of hydrazine hydrate were added into the above solution, and then the solution was heated to 90°C while stirring under an oil bath condition. After stirring for 2 h, the solution temperature was reduced to room temperature and then the Bi_2_Fe_4_O_9_ nanoparticles were added into the solution, with constant sonicating for an extra 3 h. Finally, the black mixture was washed several times with the deionized water after collecting by centrifugation and then dried in an oven at 60°C for 12 h to acquire Bi_2_Fe_4_O_9_/rGO nanohybrids. The mass ratio between Bi_2_Fe_4_O_9_ and rGO were 3:1, 2:1, and 1:1, respectively.

### Measurements of Microwave Absorption Properties

The test samples were prepared by mixing the Bi_2_Fe_4_O_9_/rGO nanohybrids with the PVDF matrix at a different weight ratio of 10, 20, and 30 wt%. The mixtures were subsequently pressed into concentric annular samples (ϕ_out_ = 7.00 mm and ϕ_in_ = 3.04 mm). To investigate the complex permittivity and permeability values from 2 to 18 GHz, an Agilent N5230C PNA-L Network Analyzer was used with a coaxial wire setup.

### Characterization

The morphologies and sizes of the Bi_2_Fe_4_O_9_ nanocubes were characterized using a scanning electron microscopy (SEM), a field emission scanning electron microscopy (FE-SEM), and an X-ray diffractometer (XRD) with CuKα radiation. To evaluate Raman spectra, a Laser Raman spectroscopy was adopted. Fourier transform infrared spectra (FT-IR) were obtained by a FT-IR spectrometer (Thermo Scientific) to observe the surface functional groups of GO and rGO. Magnetic properties of Bi_2_Fe_4_O_9_ samples were detected under normal conditions by a Vibrating Sample Magnetometer (VSM).

## Results and Discussion

**Figure 1:**
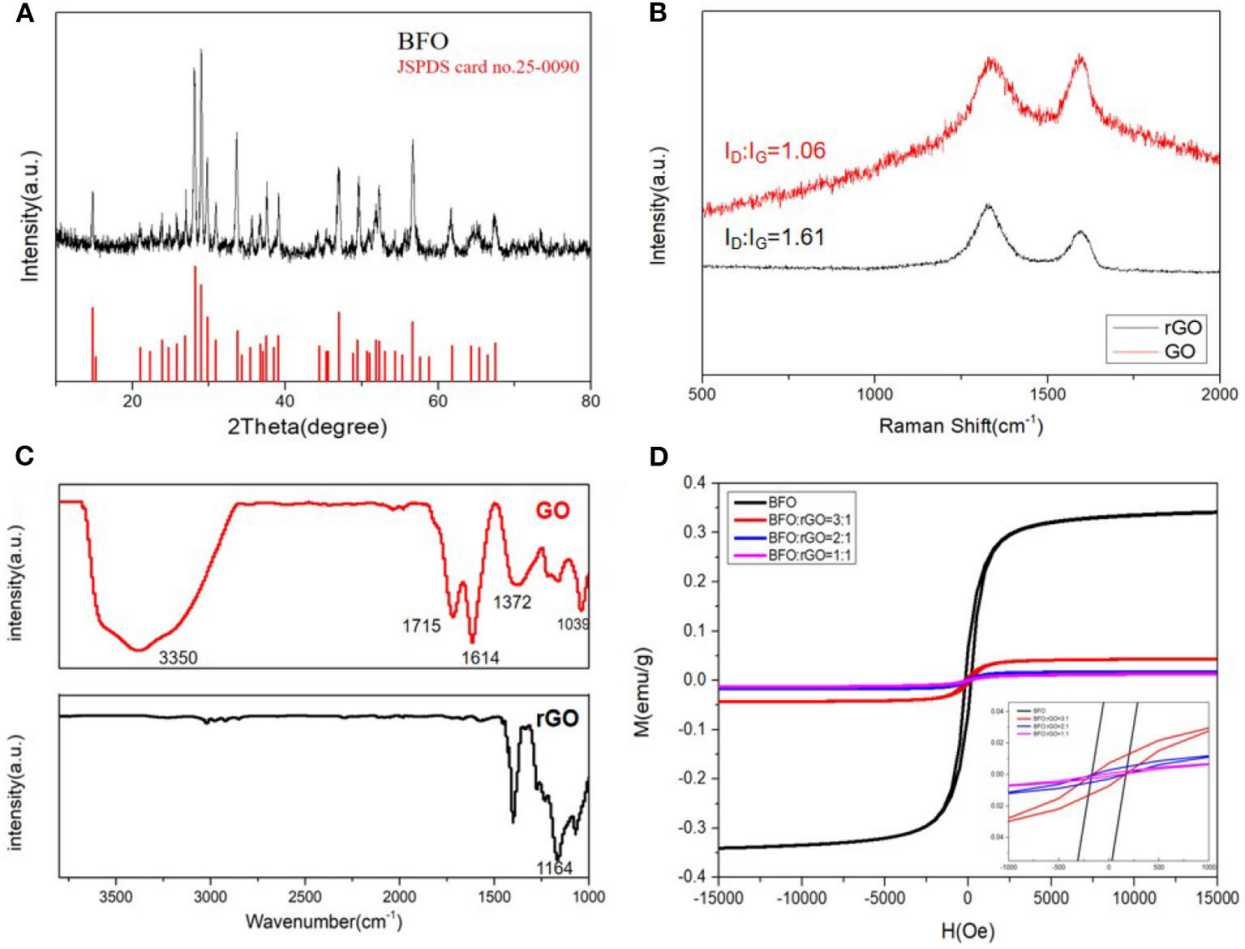
**(A)** XRD image of Bi_2_Fe_4_O_9_, **(B)** Raman spectra, **(C)** FTIR spectra of GO and rGO, **(D)** magnetic hysteresis loops of Bi_2_Fe_4_O_9_ and all kinds of ratio of BFO: rGO.

To study the crystalline structure and phase composition of the sample, the XRD pattern of the Bi_2_Fe_4_O_9_ sample can be seen in Figure 1A. The pure Bi_2_Fe_4_O_9_ nanoparticles successfully synthesized by the hydrothermal method are well-crystallized without any impurities and attribute the orthorhombic structure in accordance with the standard data (JSPDS card No.25-0090). From Figure 1B, the Raman spectrum shows the typical G band at 1,570 cm^−1^ and D band at 1,340 cm^−1^. Here, I_D_/I_G_, which presents the intensity ratio of D band (disordered carbon) to G band (sp^2^ carbon), is a common standard for determining the degree of disorder of graphitic layers (Wang et al., 2018; Xu et al., 2018b). According to the Raman spectra in Figure 1B, it is not difficult to conclude that the value of I_D_/I_G_ for rGO (1.61) is higher than the value of GO (1.06), reflecting a higher degree of in- plane defect and edge defect in the rGO due to the reduction process. These defects are closely related to the microwave absorption ability of the materials (Wang et al., 2013). The FT-IR spectrum of GO and rGO are given in Figure 1C in the range of 500–4,000 cm^−1^. For pure GO, the broad peak located at about 3,350 cm^−1^ corresponds to O-H stretching vibrations of hydroxyl groups and HOH hydrogen-bonded owing to residual water (Pan et al., 2017; Zhu et al., 2019). The characteristic peak presenting at 1,715 and 1,614 cm^−1^ represent the C = O stretching vibration of carbonyl groups and the C = C skeletal stretching vibration of aromatic carbon. As for the peak at 1,372 and 1,039 cm^−1^, this can be considered to be carboxy C-OH stretching vibration and epoxy C-O stretching vibration (Xu et al., 2018b). For rGO, it is obvious that quite a few oxygen-containing functional groups have gone, and the reduction of the hydroxyl peak has exhibited some offset according to the FT-IR spectra, indicating the high reduction of GO. And the new peaks which appeared around 1,164 cm^−1^ are the peak of secondary ammonia produced by hydrazine hydrate in the reduction process. Figure 1D, The M-H curves show that the BFO sample is a weakly magnetic substance and with the introduction of rGO, the saturation magnetization decreases further which indicates that dielectric loss plays an important role in Bi_2_Fe_4_O_9_/rGO nanohybrids.

**Figure 2:**
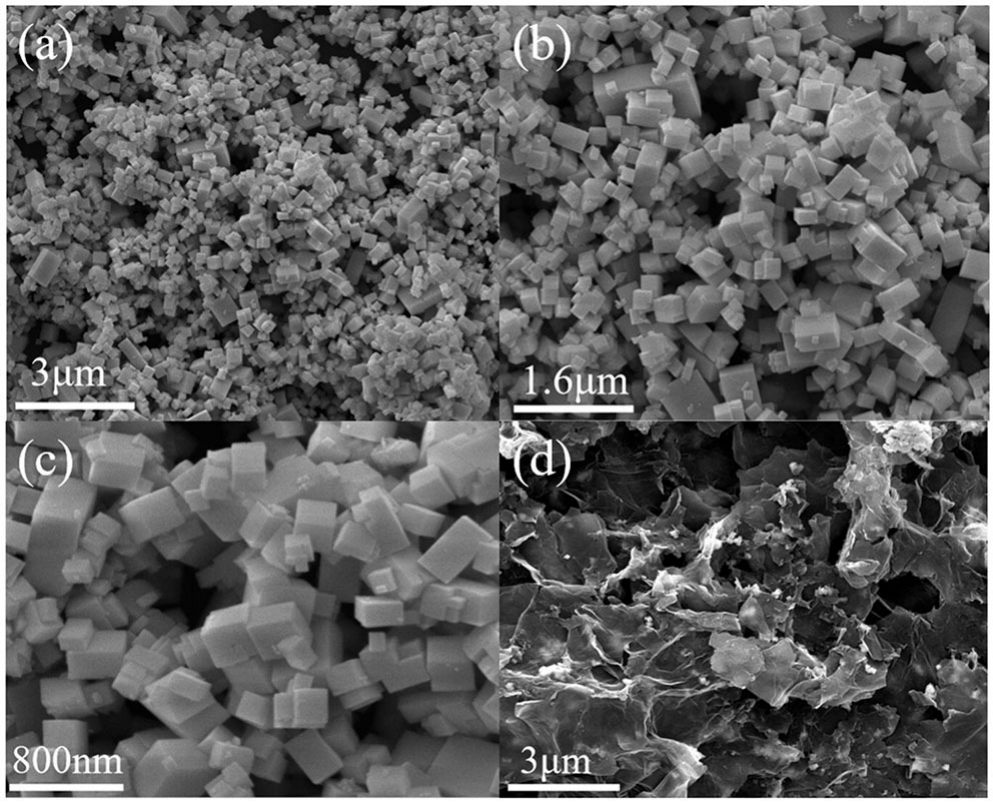
SEM image **(a,b,c)** of Bi_2_Fe_4_O_9_ nanocube, FESEM image **(d)** of Bi_2_Fe_4_O_9_/rGO nanohybrids.

To further observe the microstructure and morphology of Bi_2_Fe_4_O_9_ and Bi_2_Fe_4_O_9_/rGO nanohybrids, the SEM samples were analyzed. Figures 2a–c present the SEM image of the BFO sample, which shows the uniform bulk Bi_2_Fe_4_O_9_ cubes with an edge length of 300–800 nm. It is observed that the faces of the Bi_2_Fe_4_O_9_ structures are basically flat, though some of these cubes have a little bit of debris on their surface. Furthermore, cubic structure is the only form, and the particles are more regular and easier to prepare in large quantities. From the FESEM image (Figure 2d) for the Bi_2_Fe_4_O_9_/rGO composite, we can easily see that Bi_2_Fe_4_O_9_ cubes with an orderly pore distribution are embedded on the graphene, showing a noticeable 3D bulk-like morphology. To explore the distribution of elements, elemental mappings of Bi_2_Fe_4_O_9_/rGO nanohybrids are displayed in Figures S1c, S2. The elemental mapping images indicate that Bi, Fe, O, and C disperse homogeneously in Bi_2_Fe_4_O_9_/rGO nanohybrids. The good dispersion of these nanocubes in rGO may contribute significantly to the EM wave absorption properties.

## Microwave Absorption Performance

**Figure 3:**
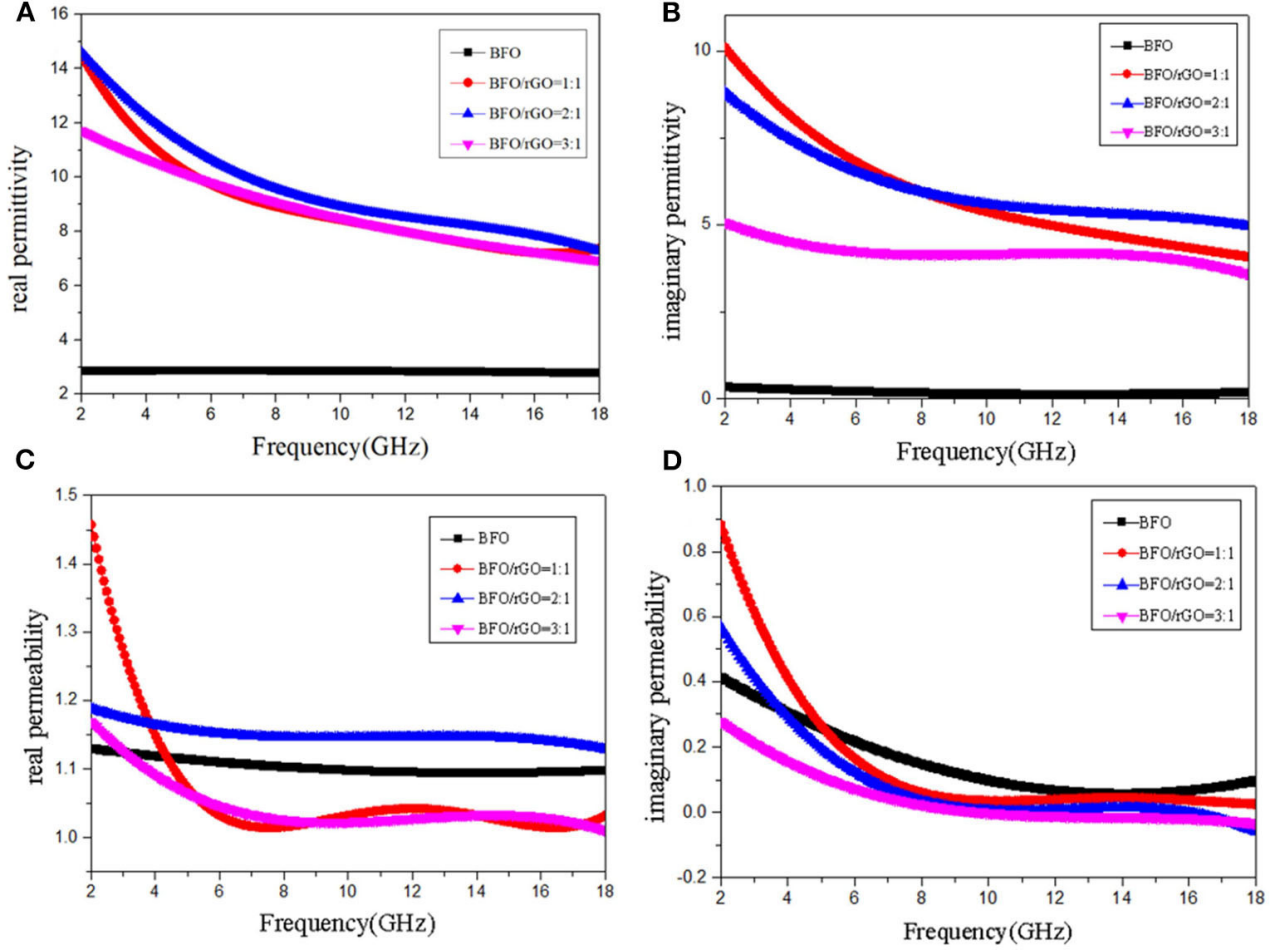
Frequency dependence of the **(A)** real part and **(B)** imaginary part of permittivity, **(C)** real part and **(D)** imaginary part of permeability of Bi_2_Fe_4_O_9_/rGO nanohybrids with different BFO ratios.

To explore the EM wave absorption performance of BFO nanocubes and BFO/rGO nanohybrids, various proportions of the products were mixed with PVDF to form the compounds via a hot-press process (Zhang et al., 2016). The complex permittivity ε_r_ (ε_r_ = ε′ - jε″) and complex permeability (μ_r_ = μ′ - jμ″) for several materials are presented in Figure 3. Among them, the complex permittivity real part ε′ represents the storage capability of electric energy and the permittivity imaginary part ε″ represents the loss capability of electric energy; complex permeability real part μ′ stands for the storage capability of magnetic energy and permeability imaginary part μ″ stands for the dissipation of magnetic energy (Zhou et al., 2017). It can be concluded that the values of ε′ and ε″ for all content in the BFO/rGO nanohybrids are much bigger than that of pure phase of BFO in Figures 3A,B. As shown in Figures 3C,D, the decline of μ′ and μ″ for BFO nanoparticles is smooth; at the same time, the decline of μ′ and μ″ for all BFO/rGO nanohybrids is also smooth from 6 to 18 GHz, but the value of μ′ and μ″ for BFO/rGO nanohybrids shows a sharp decline with increasing frequency from 2 to 6 GHz. The variation curves of the complex permittivity and complex permeability are quite untidy. We suspect this peculiar phenomenon is related to the magnetic loss mechanism.

According to the transmission line theory, the reflection loss (RL) was calculated to study the EM microwave absorption properties, where normalized input characteristic impedance (Z_in_) is calculated as:(Raghvendra et al., 2018)

(1) $$\begin{array}{l} \text{Z}\text{in}=\sqrt{\frac{\mu \text{r}}{\varepsilon \text{r}}}\tanh\left[\text{j}\left(2\text{f}\pi \text{d}/\text{c}\right)\right]\sqrt{\mu \text{r}\varepsilon }\text{r} \end{array}$$

(2) $$\begin{array}{l} R=20\text{log}\left|\frac{\text{zin-1}}{zin+1}\right| \end{array}$$

Where c is the velocity of light in free space, d is the thickness of the absorber, and f is the frequency.

**Figure 4:**
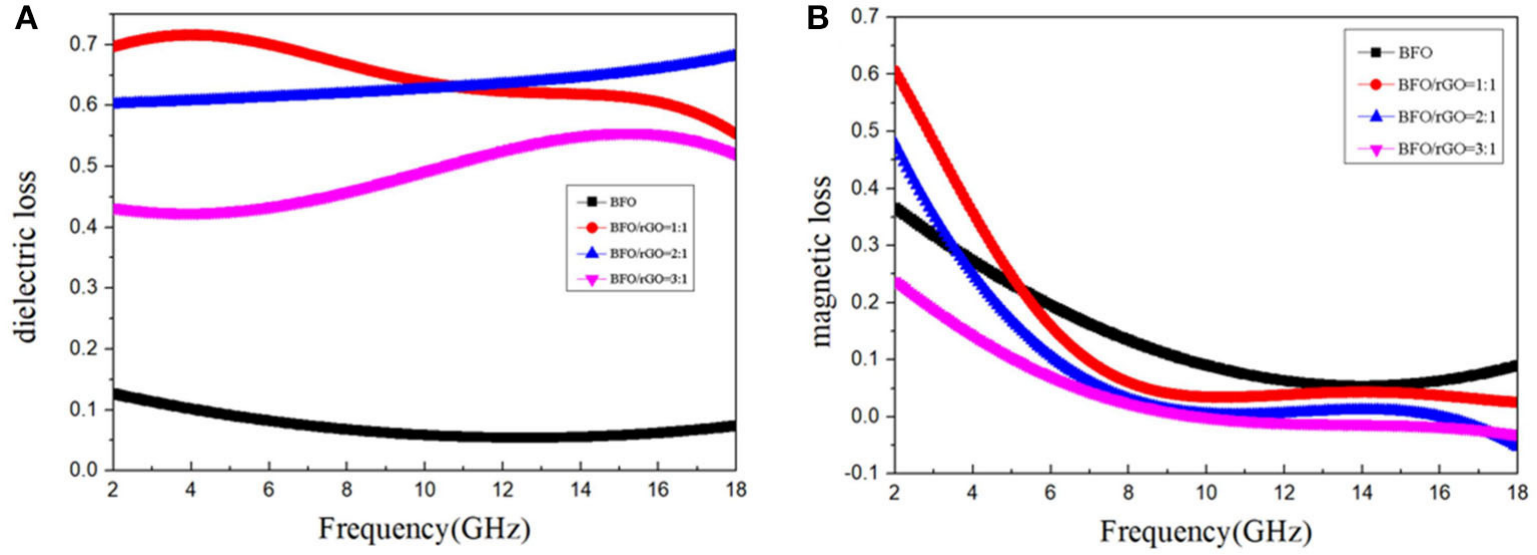
Frequency dependence of dielectric loss **(A)** and magnetic loss **(B)** of samples.

From observation, it is clearly shown that the dielectric loss values of all contents of the BFO/rGO nanohybrids are higher than their magnetic loss values in Figures 4A,B. Moreover, the dielectric loss values with the filler loading of 20 wt% BFO and 20 wt% BFO/rGO illustrate that the dielectric loss values are enhanced markedly after combining with rGO. There are two dominant common dielectric polarization mechanisms which included space charge polarization and dipolar polarization in the gigahertz frequency range. According to Maxwell-Wagner theory, interfacial polarization, which is also famous as space charge polarization occurs frequently in the composites, were composed of components with various conductivity and permittivity (Zhang et al., 2014). Compared with the weak dielectric loss performance of single BFO, rGO has an excellent dielectric loss due to the dipoles and some residual oxygen functional groups, including epoxy, hydroxyl, and carbonyl groups, which generate more polarization centers and stronger polarization relaxations(Feng et al., 2017). Apparently, BFO/rGO/PVDF composites with two kinds of interfaces produced more interfacial polarization than the single interface of the BFO/PVDF composite. Because of the existence of electrophilic fluorine in its molecular structure, PVDF is also a strong dipole material (Prasad et al., 2019). All these conditions mean BFO/rGO have higher dielectric loss values than BFO, which promoted the EM wave absorption. However, the maximum reflection loss peak is not the same frequency as that of the dielectric loss values, which indicates the main dielectric loss mechanism for BFO/rGO nanohybrids includes both dielectric loss and magnetic loss.

For a typical ferrite material, magnetic loss is usually concerned with eddy current effect, natural ferromagnetic resonance, domain wall resonance, and hysteresis (Li et al., 2018b). Generally, the domain wall resonance only operates at a megahertz frequency range and the hysteresis loss could be negligible in a weak field. Hence, the analysis of magnetic loss for BFO/rGO composites should focus on eddy current effect and natural ferromagnetic resonance. The eddy current loss is calculated by the nether equation:. If the eddy current loss is the reason for the magnetic loss, the C_0_ (C_0_ = μ″(μ′)^−2^ f^−1^) would remain constant in the corresponding frequency range (Liu et al., 2019). From the variation tendency of magnetic loss (shown in Figure 4B), it can be found that it is same with the decline of μ′ and μ”. The decline of magnetic loss for all BFO/rGO nanohybrids is smooth from 6 to 18 GHz, but the value of magnetic loss shows a sharp decline with increasing frequency from 2 to 6 GHz, which indicates that the main magnetic loss is the eddy current loss. The natural ferromagnetic resonance is related to the enhancement of anisotropic energy (Ha), which can be calculated by the following equation: (Guo et al., 2012)

(3) $$\begin{array}{l} {\text{H}}_{\text{a}}=\frac{4|{\text{K}}_{1}|}{3\mu_{0}{\text{M}}_{\text{s}}} \end{array}$$

where |K_1_| is the anisotropic coefficient and Ms is the saturation magnetization (Zhang et al., 2014). From Figure 2d, the Ms value of BFO is higher than that of BFO/rGO, which means the anisotropic energy of BFO/rGO composites is stronger. The higher anisotropic energy results in the improvement of EM absorption performance, particularly at high frequencies (Zhang et al., 2013, 2014). The results confirmed that eddy current loss and natural ferromagnetic resonance play a common role in regulating the magnetic loss of the BFO/rGO sample.

**Figure 5:**
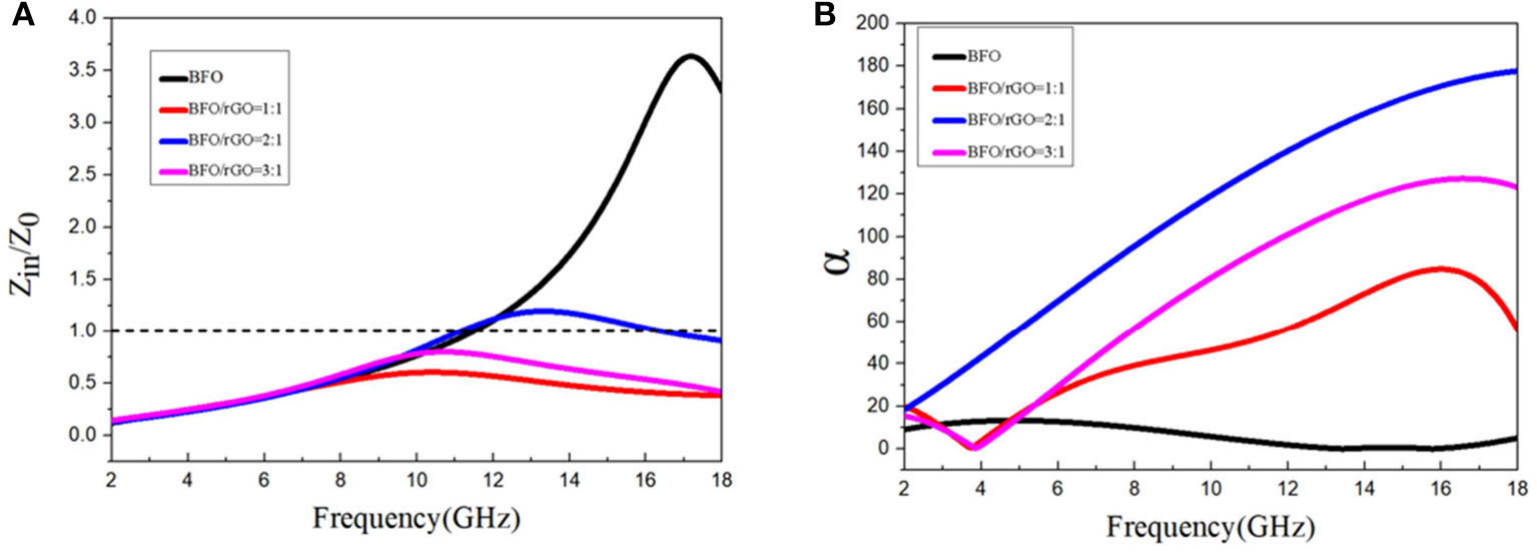
The frequency dependence of **(A)** |*Z*in/*Zo*| and **(B)** attenuation coefficient (α) for BFO nanocube and Bi_2_Fe_4_O_9_/rGO nanohybrids with a Bi_2_Fe_4_O_9_/rGO ratio of 1:1, 2:1, and 3:1.

It is well-known that a good electromagnetic wave absorber must satisfy the two conditions of impedance matching and attenuation characteristic. Impedance matching ratio can be easily understood, as the incident electromagnetic wave can be propagated to the efficient absorber and be converted into heat energy or dissipation through interference, rather than reflecting directly on the surface of absorbers (Chen et al., 2017; Fang et al., 2017; Li et al., 2018c; Xu et al., 2019). The |*Z*in/*Zo*| value, which can be calculated by the above Equation (1), shows impedance matching performance (Chen et al., 2017). The frequency dependence of the |*Z*in/*Zo*| value (d = 2.4 mm) of various samples can be observed in Figure 5A. Compared with the value of sample BFO, the value of sample BFO/rGO with a ratio of 2:1 is close to 1 in the high frequency region, which means that it has a relatively good impedance matching in the corresponding frequency range. Through Figure 5A, it is also proven that it is possible to get a better impedance matching by introducing rGO and changing the ratio of BFO and rGO.

Another factor which is associated with a desirable electromagnetic wave absorption property is electromagnetic attenuation capability, which can be represented by attenuation constant α on the following equation: (Xiang et al., 2014)

(4) $$\begin{array}{l} \alpha =\frac{\sqrt{2}\pi \text{f}}{\text{c}}\times \sqrt{\left(\mu^{,,}\varepsilon^{,,}-\mu^{,}\varepsilon^{,}\right)+\sqrt{{\left(\mu^{,,}\varepsilon^{,,}-\mu^{,}\varepsilon^{,}\right)}^{2}+{\left(\mu^{,}\varepsilon^{,,}-\mu^{,,}\varepsilon^{,}\right)}^{2}}} \end{array}$$

**Figure 6:**
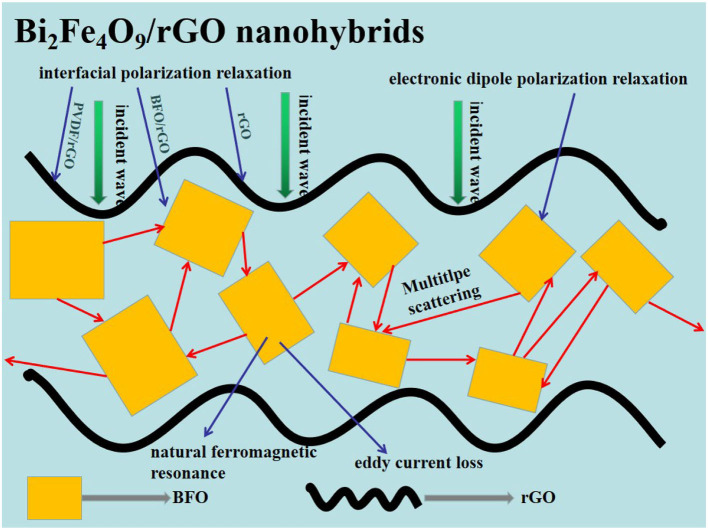
Illustration of microwave wave absorption mechanisms of the Bi_2_Fe_4_O_9_/rGO nanohybrids.

Where f is the frequency of the EMW and c is the velocity of light. Hence, excellent microwave absorption performance is related to the combination of impedance matching and high attenuation ability. According to the different ratios of BFO and rGO, adjusting the appropriate basic electromagnetic parameters is conducive to improving the microwave absorption performance. As shown in Figure 5B, because the high dielectric loss and eddy current loss in high frequency, the α value of BFO/rGO samples show an increase trend which BFO samples do not have with increasing frequency. It is also obvious to see the attenuation coefficient value of sample BFO/rGO with a ratio of 1:1 is pretty high; however, it does not have a good microwave absorption performance due to its bad impedance matching. Therefore, the combination of impedance matching and high attenuation ability is an effective way to get an excellent microwave absorption performance as shown in Figure 6. The suitable fundamental electromagnetic parameters are adjusted by changing the contents of the BFO and rGO, which are beneficial to enhancing microwave absorption performance.

**Figure 7:**
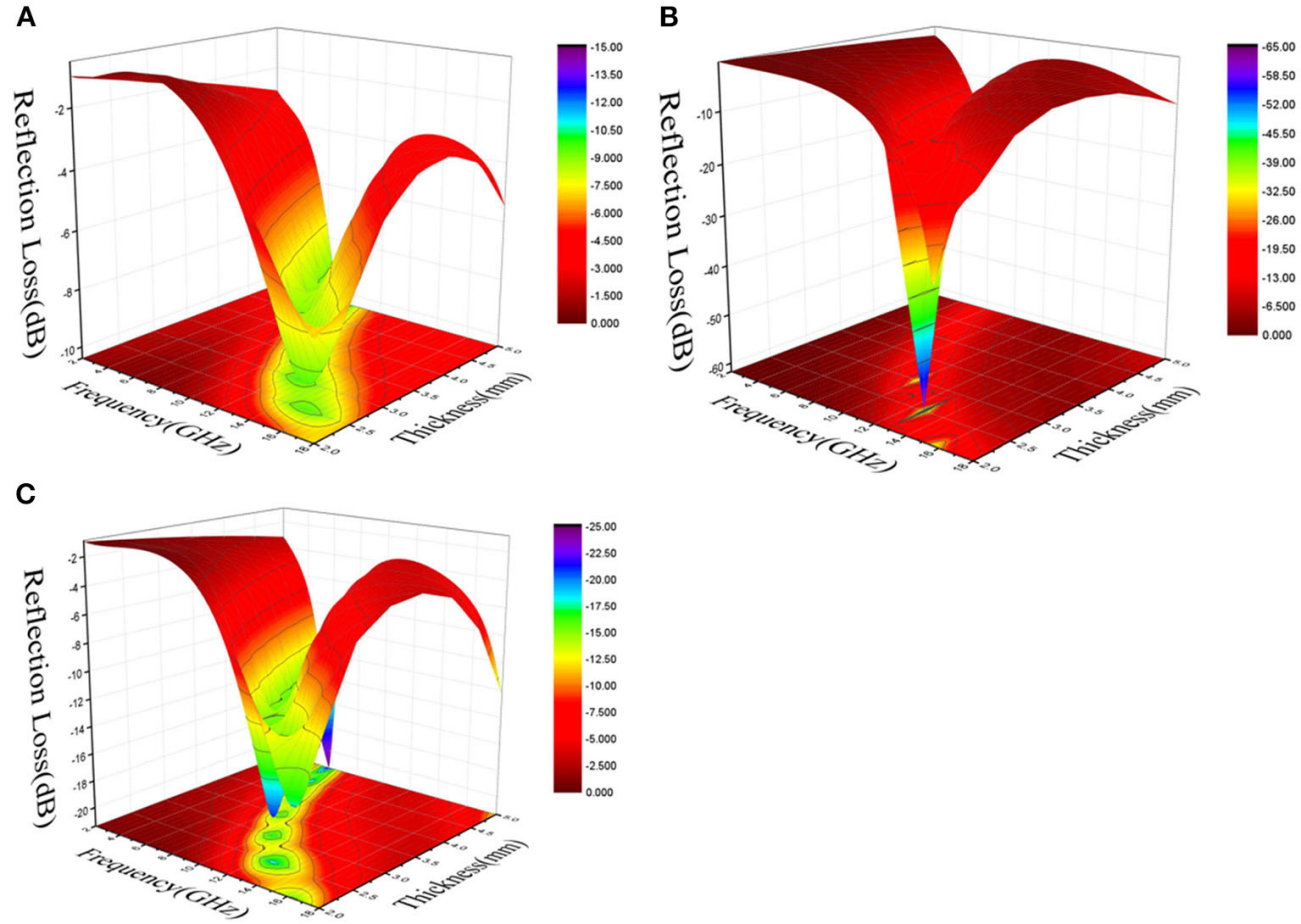
3D image maps of the reflection loss of all composites (*w* = 10 wt %). **(A,B,C)** for Bi_2_Fe_4_O_9_/rGO nanohybrids with a Bi_2_Fe_4_O_9_/rGO ratio of 1:1, 2:1, and 3:1.

**Figure 8:**
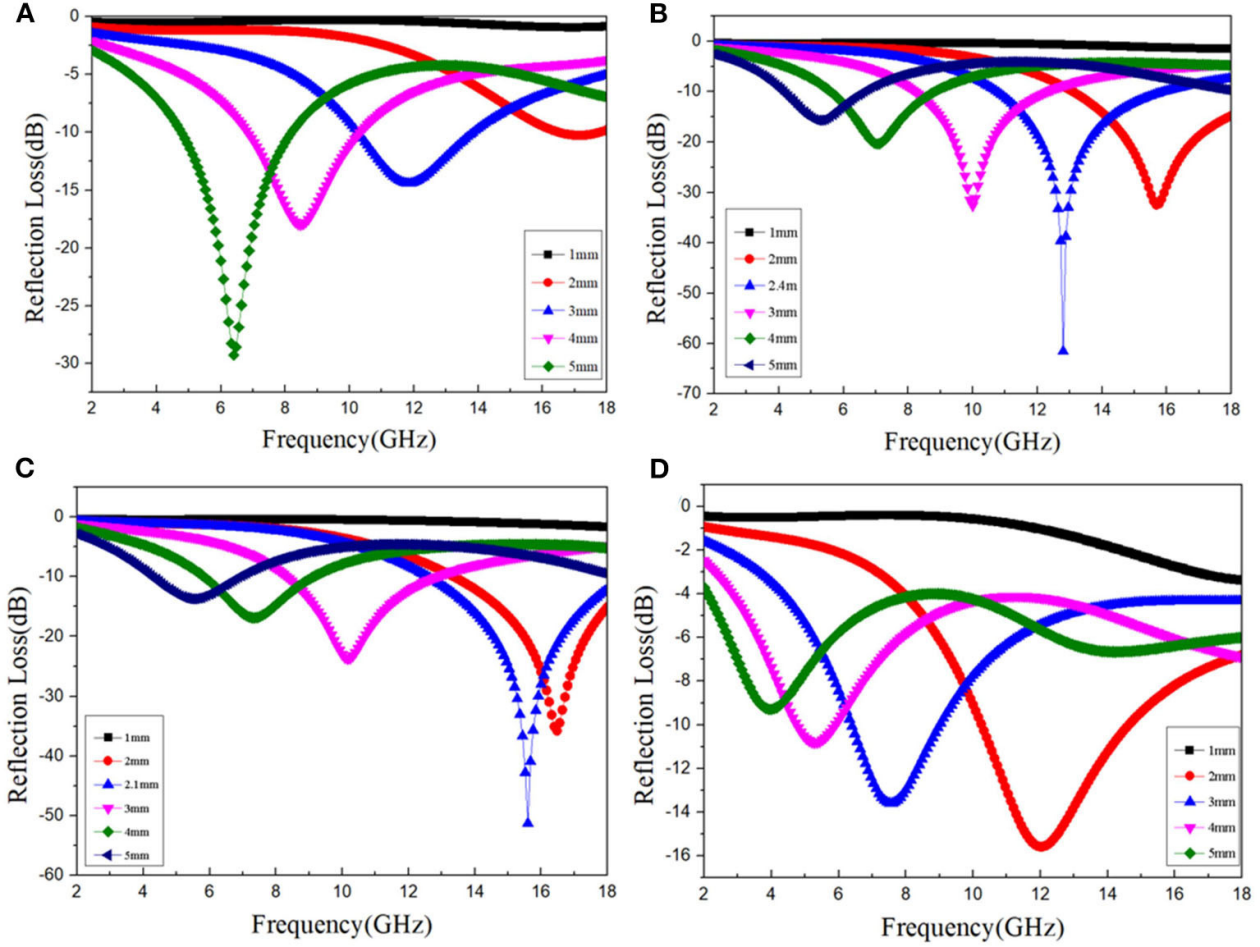
RL curves of the composites with the thickness of 1–5 mm in the frequency range of 2–18 GHz for Bi_2_Fe_4_O_9_/rGO nanohybrids with Bi_2_Fe_4_O_9_/rGO ratio of 2:1 under different filler loading. **(A)**: 5% wt, **(B)**: 10% wt, **(C)**: 15% wt, **(D)**: 20% wt.

Figures 7A–C show the three-dimensional images of calculated theoretical RLs of the BFO/rGO nanohybrids at different thicknesses (1–5 mm) in the frequency range of 2–18 GHz with the filler loading of 10 wt% with the ratio of BFO/rGO, 1:1, 2:1; and 3:1, respectively. Meanwhile, it is not hard to see that we can regulate the ability of BFO/rGO composites to absorb electromagnetic waves by adjusting the ratio of the BFO and rGO. Since the reflection loss properties are very sensitive to the content of rGO in BFO/rGO nanohybrids, Figure 8 shows the calculated RLs for the BFO/rGO absorber with thicknesses varying from 1 to 5 mm in the frequency range of 2–18 GHz with the filler loading of 5, 10, 15, and 20 wt% with a BFO/rGO ratio of 2:1. Compared with Figure 8 and Figure S3, with the introduction of rGO, the microwave absorption performances of composites are largely enhanced. Meanwhile, all the minimal RL values are < -10 dB in Figures 8A–C with thicknesses of 2–5 mm. When the ratio of BFO/rGO is 1:1, optimal RL value reaches −40 dB at a pretty high frequency with a relatively small bandwidth in Figure S4c, but the minimum RLs gradually turn to a low frequency range with the increasing thickness. The effective absorption bandwidth below −10 dB for the three absorbers can cover 4.0–18.0 GHz in Figure 8C and Figures S4c, S5c, respectively. From Figure 8B, one outstanding microwave absorption property with an optimal RL value of −61.5 dB and a broad effective bandwidth of 5 GHz (10.8–15.8 GHz), and a thin matched thickness of 2.4 mm, is achieved with the ratio BFO/rGO of 2:1. It is not difficult to see that the microwave absorbing ability of the BFO/rGO nanohybrids at various frequencies can be regulated by changing the ratio of the rGO and BFO through Figure 8 and Figures S4, S5. Furthermore, we can conclude that, with the increase of thicknesses of 2–5 mm in Figure 8 and Figures S4, S5, the maximum peak value develops to a low frequency when increasing the thickness, which indicates BFO/rGO/PVDF composites will become a potential excellent microwave absorption material.

## Conclusion

We have synthesized three-dimensional bulk Bi_2_Fe_4_O_9_ nanocubes using a simple hydrothermal method, and the Bi_2_Fe_4_O_9_ nanoparticles successfully loaded on graphene forming a BFO/rGO/PVDF composite absorber. It is proven that it is a positive way to obtain a potential EMW material by combining BFO, rGO, and PVDF. Specifically, the introduction of rGO sheets and PVDF dramatically ameliorated the impedance matching of BFO because of the synergy effect between multiple components. When the ratio of Bi_2_Fe_4_O_9_ to rGO reaches 2:1 with an absorber thickness of 2.4 mm, the composite reaches −61.5 dB at 12.8 GHz, possessing a rather wide frequency band of 10.8–15.8 GHz (RL < -10 dB). Moreover, the thickness of the absorber is a pivotal factor in practical applications, meaning that Bi_2_Fe_4_O_9_/rGO nanohybrids are very significant for developing thin EM wave absorbing materials. Therefore, the composite has a broad application prospect in the field of microwave absorption.

## Data Availability Statement

The raw data supporting the conclusions of this article will be made available by the authors, without undue reservation.

## Author Contributions

ML and Y-KS: data curation and writing- original draft preparation. S-HY and H-YW: conceptualization, methodology, visualization, and investigation. X-HG and G-SW: supervision. All authors contributed to the article and approved the submitted version.

## Conflict of Interest

The authors declare that the research was conducted in the absence of any commercial or financial relationships that could be construed as a potential conflict of interest.
