# Supplementary material for: Three-Dimensional Bi2Fe4O9 Nanocubes Loaded on Reduced Graphene Oxide for Enhanced Electromagnetic Absorbing Properties
Source: Front Chem. 2020 Aug 7;8:608. doi: 10.3389/fchem.2020.00608 (PMC7427411; doi:10.3389/fchem.2020.00608)

Supporting Information

Three-dimensional $Bi_2Fe_4O_9$ Nanocubes Loaded on Reduced Graphene Oxide for Enhanced Microwave Absorption

Min Lu[1], Yuan-Kai Sun[1], Shu-Hao Yang[2], Hui-Ya Wang[2], Xiao-Hui Guan[1]*, Xiao-Bo Sun[2]* and Guang-Sheng Wang[2]*

[1]School of Chemical Engineering, Northeast Electric Power University, Jilin 132000, China; E-mail: guanxh@neepu.edu.cn (Xiao-Hui Guan)

[2]School of Chemistry, Beihang University, Beijing 100191, PR China

[*] E-mail: sunxb@buaa.edu.cn (Xiao-Bo Sun) and wanggsh@buaa.edu.cn (Guang-Sheng Wang);

Supplementary Figures

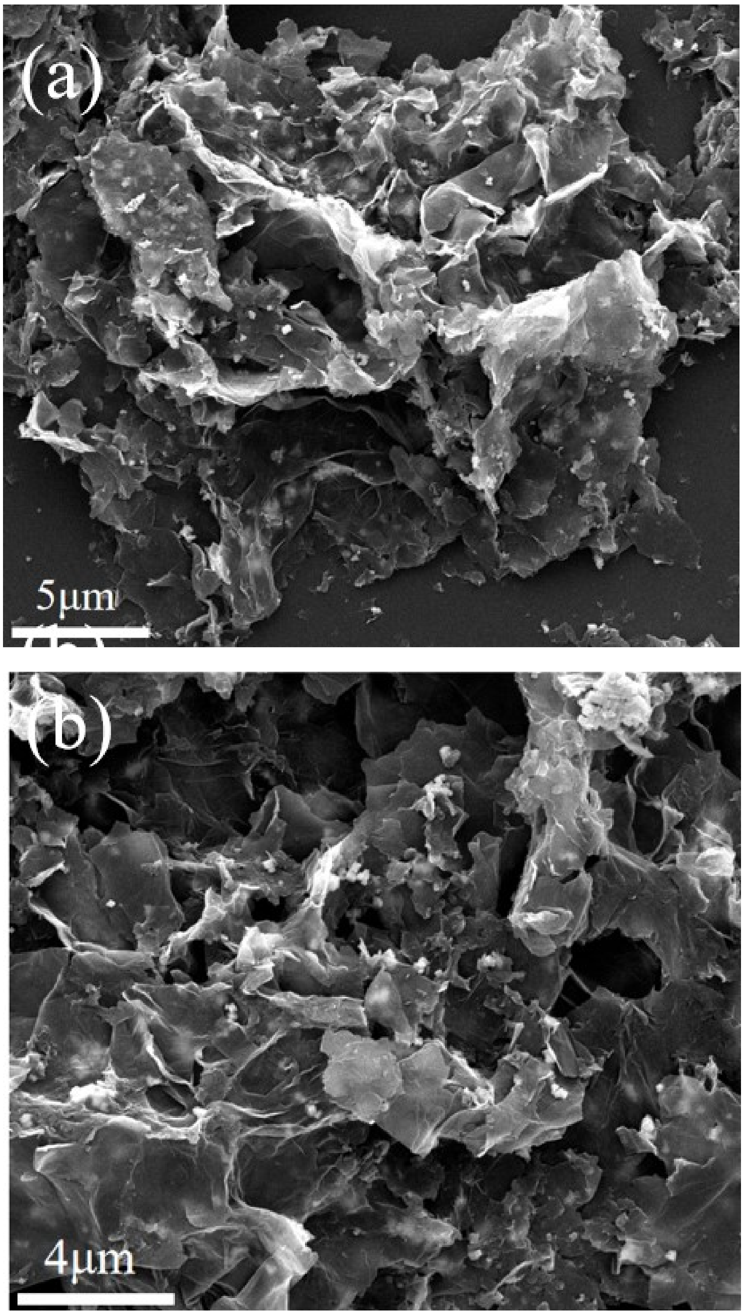

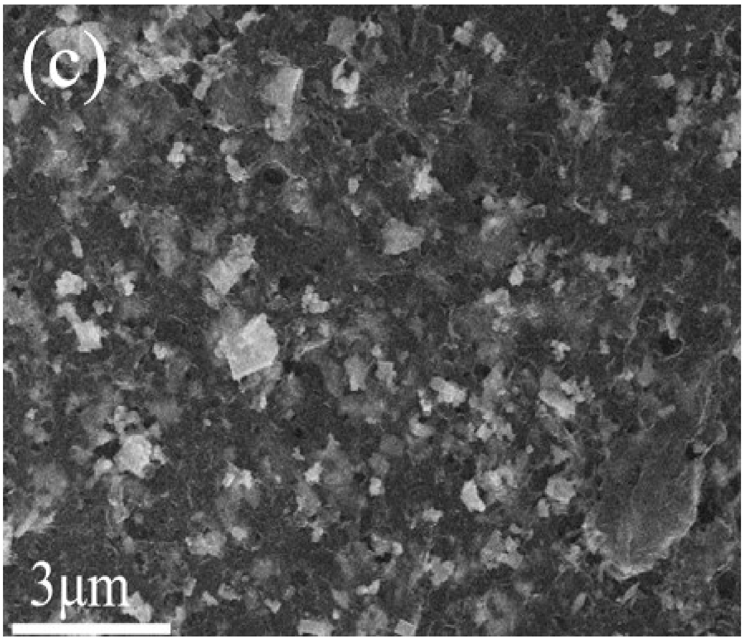

Figure S1. FESEM image of the $Bi_2Fe_4O_9$/rGO nanohybrids

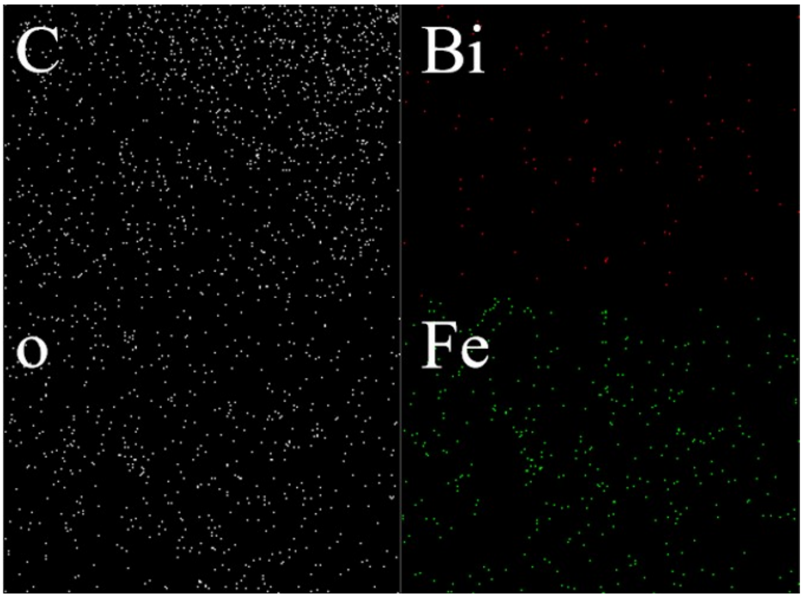

Figure S2. Elemental mapping images corresponded with $Bi_2Fe_4O_9$/rGO nanohybrids of C, Bi, O and Fe.

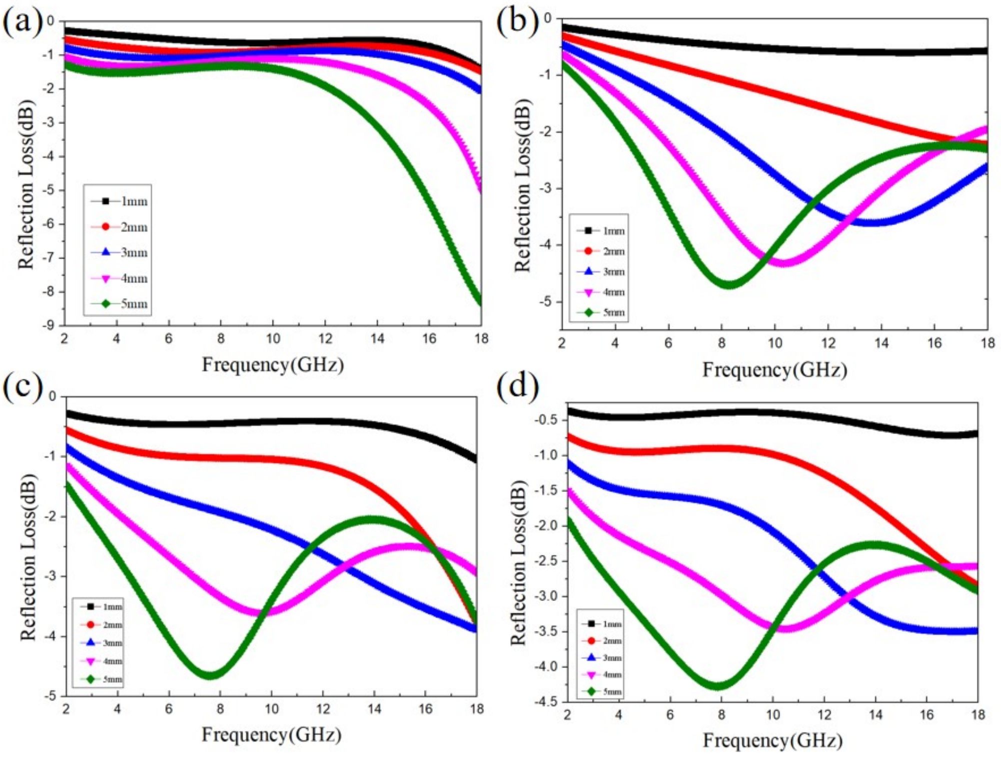

Figure S3. RL curves of the composites with the thickness of 1 mm~5 mm in the frequency range of 2–18 GHz for pure $Bi_2Fe_4O_9$ cubes under different filler loading, (a):5% wt, (b):10% wt, (c):15% wt, (d):20% wt.

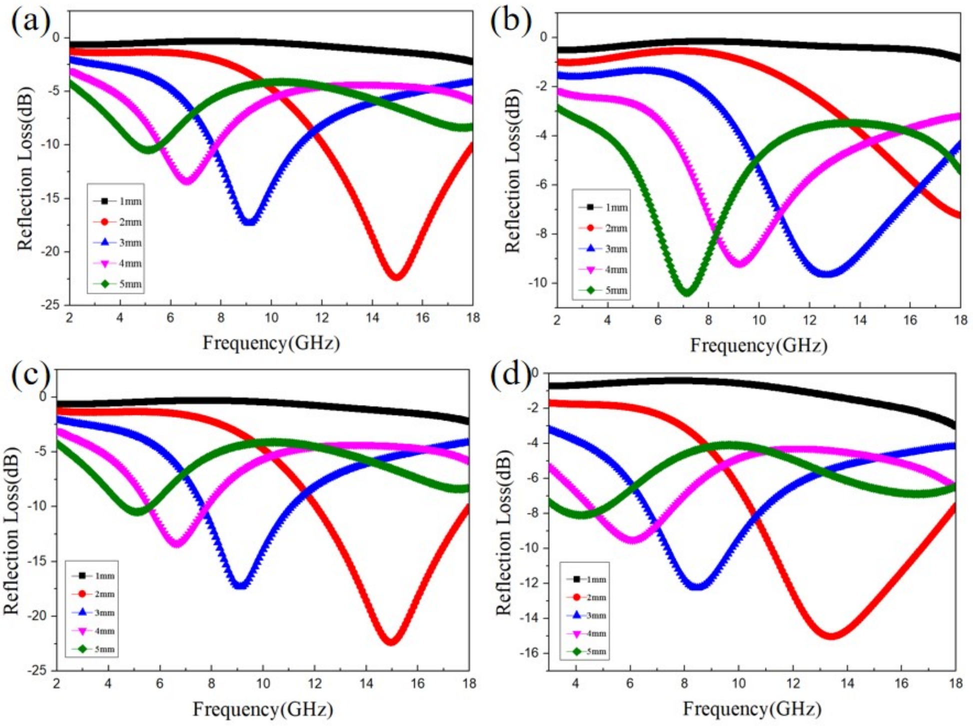

Figure S4. RL curves of the composites with the thickness of 1 mm~5 mm in the frequency range of 2–18 GHz

for $Bi_2Fe_4O_9$/rGO nanohybrids with $Bi_2Fe_4O_9$/rGO ratio of 1:1 under different filler loading. (a):5% wt, (b):10% wt, (c):15% wt, (d):20% wt.

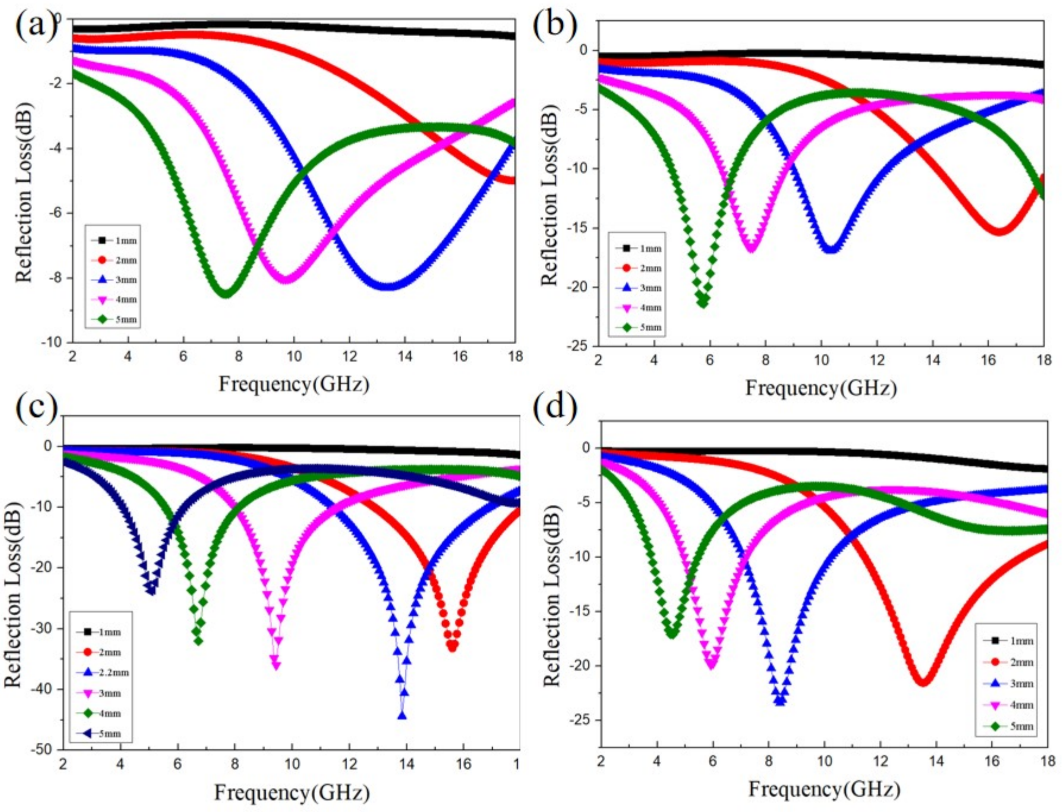

Figure S5. RL curves of the composites with the thickness of 1 mm~5 mm in the frequency range of 2–18 GHz for $Bi_2Fe_4O_9$/rGO nanohybrids with $Bi_2Fe_4O_9$/rGO ratio of 3:1 under different filler loading. (a):5% wt, (b):10% wt, (c):15% wt, (d):20% wt.

Graphical Abstract

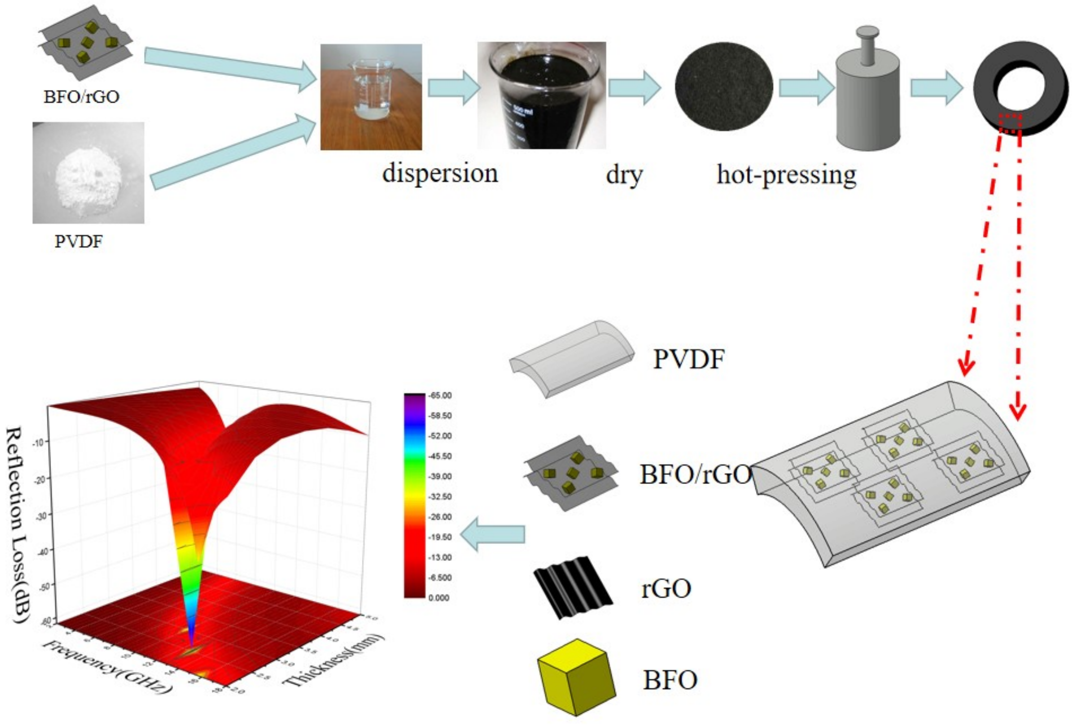

Supplement: Supplementary file 1 [file Table_1.pdf]
